# Oral viral, fungal, and bacterial infections linked to comorbidities: A case series from a Brazilian referral center

**DOI:** 10.4317/jced.62619

**Published:** 2025-04-01

**Authors:** Daniella Estanho, Lucas Fellipe do Amaral-Sobrinho, Fernanda Silva de Lima, João Pedro Silva Contreiras, Michelle Agostini, Natália Silva Andrade, José Alcides Almeida de Arruda, Sandra R. Torres, Sílvia Paula de Oliveira, Bruno Augusto Benevenuto de Andrade, Jefferson R. Tenório

**Affiliations:** 1 Department of Oral Diagnosis and Pathology, School of Dentistry, Universidade Federal do Rio de Janeiro, Rio de Janeiro, Brazil; 2 Department of Dentistry, School of Dentistry, Universidade Federal de Sergipe, Sergipe, Brazil; 3 Clinical Dentistry Service, Hospital Universitário Clementino Fraga Filho, Universidade Federal do Rio de Janeiro, Rio de Janeiro, Brazil

## Abstract

**Background:**

Oral infections exhibit variability in their causative agents and clinical presentations, underscoring the necessity of accurate diagnosis for effective management. Despite extensive documentation globally, data on these infections from Brazil remain scarce. This study aimed to assess the occurrence, clinical features, and differential diagnosis of oral viral, fungal, and bacterial infections at a single center in southern Brazil.

**Material and Methods:**

A retrospective analysis was conducted between 2010 and 2023. Clinicodemographic data, comorbidities, and routine medication use were analyzed descriptively and statistically.

**Results:**

A total of 462 cases were included. The median age was 49.5 years (range: 2–100). Viral infections were the most frequent (65.8%), with squamous papilloma accounting for 49.4% of cases. Fungal infections comprised 29.4% of cases, predominantly erythematous candidiasis (20.8%) and pseudomembranous candidiasis (5.6%). These infections were more common in women, older adults (*p*<0.001), and individuals with comorbidities such as systemic arterial hypertension (*p*=0.006) and diabetes mellitus (*p*=0.028). Bacterial infections were rare (4.8%), with actinomycosis being the most frequent (2.2%).

**Conclusions:**

Data from our series on oral viral, fungal, and bacterial infections align with the literature. The results emphasize the importance of tailored diagnostic approaches, particularly for at-risk patient populations.

** Key words:**Bacterial infections, Communicable diseases, Mycoses, Oral manifestations, Virus diseases.

## Introduction

Oral and maxillofacial structures are frequently affected by a wide range of infectious processes, with odontogenic infections being the most prevalent in clinical practice (1). However, these regions are also commonly impacted by nonodontogenic infections, including those of bacterial, viral, and fungal origin (2-4). The incidence of these infections varies globally and is influenced by factors such as age, immune status, and geographic location. For instance, herpes simplex virus type 1 (HSV-1) is the primary cause of oral herpes, with approximately 3.8 billion people under the age of 50 infected with oral or genital HSV-1 (5). Similarly, oral candidiasis is the most common fungal infection of the oral cavity, particularly among immunocompromised individuals, such as those living with HIV/AIDS or undergoing antineoplastic therapy (6). In addition to periodontitis, the most widespread bacterial infection in the oral cavity, nonodontogenic bacterial infections (e.g., syphilis and tuberculosis) have shown increasing prevalence and continue to pose significant health threats (2,7,8).

Oral infections present a diverse array of clinical manifestations, requiring detailed examination and various diagnostic tools, including serological testing, exfoliative cytology, and histopathology (2,8,9). While common infections such as candidiasis and herpes labialis are frequently encountered in clinical settings (10,11), more complex chronic invasive infections—such as paracoccidioidomycosis and histoplasmosis—pose significant diagnostic challenges (2,12,13). These infections can range from asymptomatic presentations to systemic symptoms such as pain, fever, and malaise, significantly impairing patients’ quality of life (7,8). Moreover, their diverse pathophysiologies and the rarity of certain infections often result in diagnostic delays, leading to advanced disease states and increased transmission risks. Consequently, clinicians must remain well-informed to enable timely diagnosis and effective intervention (2).

Local immunity factors, such as hyposalivation and prolonged use of topical corticosteroids, predispose individuals to certain oral infections (14,15). Additionally, systemic health conditions play a critical role in susceptibility to nonodontogenic infections. Individuals with uncontrolled diabetes mellitus, HIV/AIDS, hematological disorders, nutritional deficiencies, or those undergoing immunosuppressive therapy are at a heightened risk for severe and refractory infections (16). In such cases, oral lesions may serve as early indicators of significant underlying systemic conditions (17).

Although numerous studies have documented nonodontogenic infections, most focus on isolated types rather than offering a comprehensive epidemiological analysis of multiple types (7,13,18). This contributes to the relative scarcity of data on these infections in South America. As far as we know, no Brazilian study has comprehensively analyzed the profile of individuals with nonodontogenic infections. Therefore, the purpose of the present study was to evaluate the occurrence and clinical profile of oral lesions associated with viral, fungal, and bacterial infections based on data collected over a 13-year period at a single referral center in Brazil.

## Material and Methods

-Case series and ethical clearance

A retrospective analysis was conducted between 2010 and 2023 in the Department of Pathology and Oral Diagnosis at the Universidade Federal do Rio de Janeiro, Rio de Janeiro, Brazil. The study adhered to the Strengthening the Reporting of Observational Studies in Epidemiology (STROBE) guidelines (19). Ethics approval was obtained from the Institutional Research Ethics Committee (Approval No. 6180132), and patient confidentiality was maintained in compliance with the principles of the Declaration of Helsinki.

-Eligibility criteria and diagnostic rendering

Records of individuals diagnosed with viral, fungal, or bacterial infections in the oral cavity were included, irrespective of sex or age, provided the diagnosis was confirmed through clinical information and complementary examinations. Diagnoses of fungal infections were primarily based on histopathological analysis and specific histochemical staining methods, including periodic acid-Schiff (PAS) and Gomori methenamine silver (GMS) stains. Viral infections were confirmed through serological testing and, when necessary, cytological examinations. Bacterial infections were identified based on clinical presentation and confirmed through culture testing or therapeutic response to antibiotics (20). All diagnoses were rendered by two oral medicine specialists (M.A. and J.R.T.). Cases lacking sufficient diagnostic information or with incomplete clinical follow-up were excluded. Odontogenic infections and orofacial space infections (e.g., pulpal/apical diagnoses and Ludwig’s angina) were beyond the scope of this study, as the service does not provide urgent/emergency care.

-Data collection

Data were collected by three trained authors (D.E., L.F.A.S., and F.S.L.) and included the following variables: age, sex, type of infectious disease (viral, fungal, and bacterial), evolution time (in months), anatomical site of the lesion, presence of symptoms, clinical pattern of oral lesions (e.g., nodule, papule, erythema, plaque), comorbidities, and use of routine medications.

-Data analysis

Statistical analysis was performed using the Statistical Package for the Social Sciences (SPSS) software (IBM SPSS Statistics for Windows, version 27.0, IBM Corp., Armonk, NY, USA). The Kolmogorov-Smirnov test was used to assess the normality of quantitative variables, confirming a non-parametric distribution. Descriptive analysis was performed for all variables. For bivariate analysis, the Kruskal-Wallis test and Dunn post hoc test were used to compare quantitative variables with independent variables. Associations between sociodemographic factors, anatomical site, clinical signs/symptoms, and types of infectious diseases were assessed using Pearson’s Chi-Square and Likelihood Ratio tests. For all analyses, the level of significance was set at *p*<0.05.

## Results

**Figure 1:**
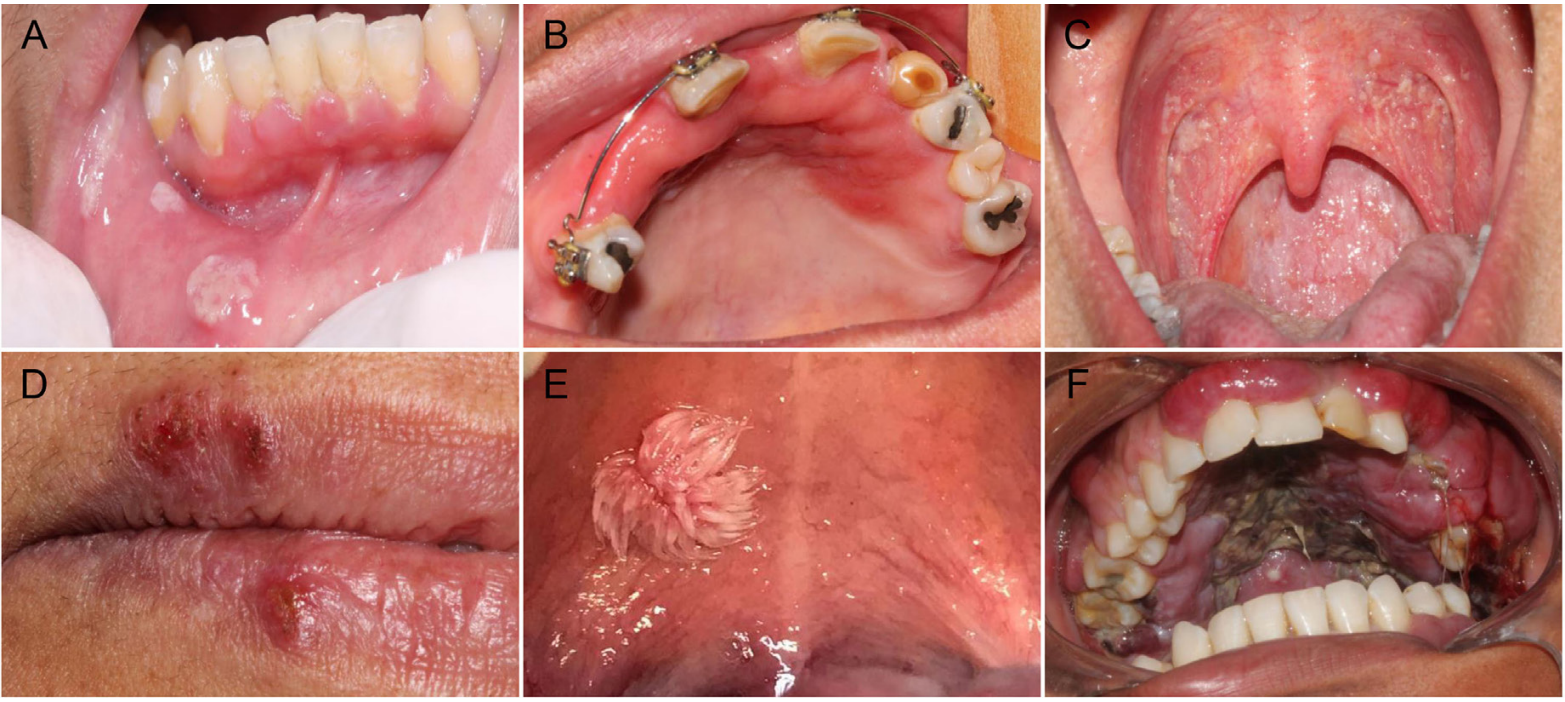
Clinical presentations of viral, fungal, and bacterial infections of the oral cavity. (A) Secondary stage of acquired oral syphilis, presenting as multiple rounds, grayish-white mucous patches on the lower right labial mucosa. (B) Erythematous candidiasis appears as diffuse erythema on the hard palate and gingiva. (C) Pseudomembranous candidiasis, characterized by white, removable plaques with an underlying erythematous base on the oropharyngeal mucosa. (D) Herpes labialis displayed as clustered, crusted lesions with erythematous borders and ulceration on the right lower lip and the transition between the skin and the semi-mucosa of the upper lip. (E) Squamous papilloma appearing as a solitary, exophytic lesion with a verrucous (“cauliflower-like”) appearance on the right soft palate. (F) Kaposi’s sarcoma manifesting as a red-purple, exuberant, multilobulated tumor and patches with irregular borders on the hard palate and gingiva.

A total of 462 oral infections were diagnosed, comprising viral (n=304; 65.8%), fungal (n=136; 29.4%), and bacterial (n=22; 4.8%) cases. Most infections occurred in individuals in their fifth decade of life, with a median age of 49.5 years (range: 2–100), and a predominance of females (n=260; 56.3%). Among viral infections, human papillomavirus (HPV)-related lesions were the most common, with squamous papilloma (n=228; 75%) being the predominant type, followed by verruca vulgaris (n=23; 7.6%) and herpes simplex virus lesions (secondary herpes labialis) (n=17; 5.6%). Fungal infections were predominantly erythematous candidiasis (n=96; 70.6%) and pseudomembranous candidiasis (n=26; 19.1%). Actinomycosis (n=10; 45.4%) was the most frequent bacterial infection ( Table 1). Representative examples of viral, fungal, and bacterial infections in the oral cavity are illustrated in Figure 1.

**Figure 2:**
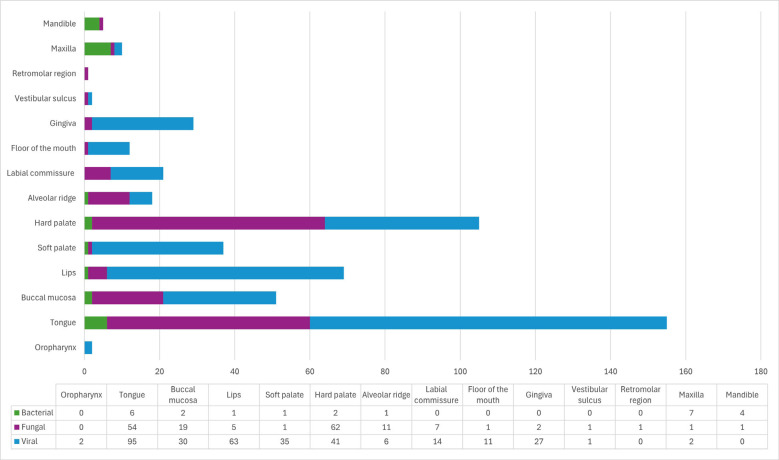
Frequency of anatomical locations of individuals with oral infectious diseases (n=462).

**Figure 3:**
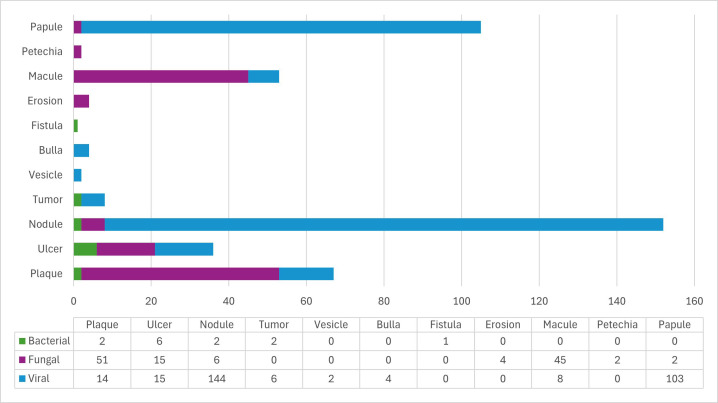
Frequency of clinical patterns of oral infectious diseases (n=462).

**Figure 4:**
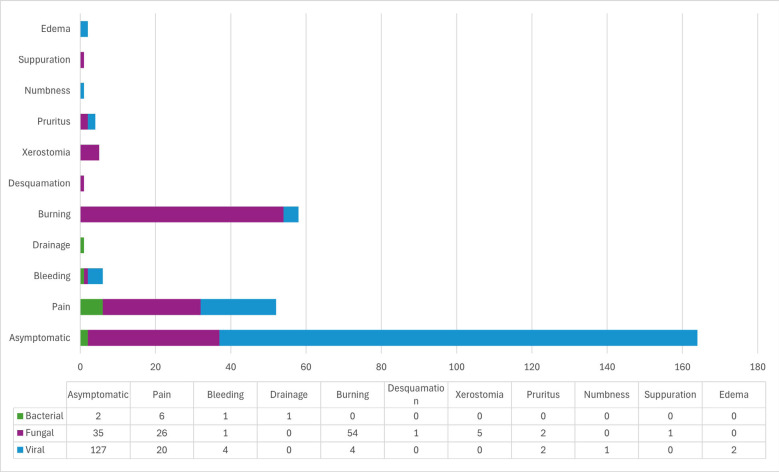
Frequency of signs and symptoms of individuals with oral infectious diseases (n=462).

Fungal infections were primarily located on the hard palate (n=62; 59.0%) and tongue (n=54; 34.8%), while viral infections most frequently affected the tongue (n=95; 61.3%). Bacterial infections were predominantly found in the maxilla (n=7; 70.0%) (Fig. 2). Clinically, fungal infections commonly presented as plaques (n=51; 40.8%) or macules (n=45; 36%) and were significantly associated with a burning sensation (n=54; 43.2%, *p*<0.001) (Figs. 3,4,  Table 2). Viral infections were predominantly nodular (n=144; 48.6%, *p*<0.001) or papular (n=103; 34.8%, *p*<0.001) and were mostly asymptomatic (n=127; 79.4%) (Figs. 3,4). Bacterial infections primarily manifested as ulcerated lesions with exposed bone (n=6; 46.1%, *p*<0.001) and were often painful (n=6; 60%, *p*<0.001) (Figs. 3,4,  Table 2).

Fungal infections were significantly more common in females (*p*<0.001), older adults (*p*<0.001), and individuals who consumed alcohol (*p*=0.038). Viral infections, in contrast, were more frequently associated with males (*p*=0.001). There were no significant differences in mean duration of lesions among bacterial (5.4 months), fungal (6.8 months), and viral (16 months) infections (*p*>0.05) ( Table 3). Fungal infections were significantly associated with the hard palate (59.0%, *p*<0.001) and alveolar ridge (61.1%, *p*=0.014), while viral infections were more common on the gingiva (93.1%, *p*<0.001), lips (91.3%, *p*<0.001), and soft palate (94.6%, *p*<0.001). Although bacterial infections were less frequent, they showed a significant association with the maxilla (70.0%, *p*<0.001) and mandible (80.0%, *p*<0.001).

Data on comorbidities and routine medication use were available for 177 records. The most common comorbidities were systemic arterial hypertension (n=66; 37.3%), diabetes mellitus (n=28; 15.8%), and hypothyroidism (n=16; 9.0%) ( Table 4). Systemic arterial hypertension and diabetes mellitus were both significantly associated with fungal infections (*p*=0.006 and *p*=0.028, respectively), while viral infections were more frequently observed in individuals living with HIV (*p*=0.013). The most commonly used medications were antihypertensives (n=65), gastric protectors (n=27), oral hypoglycemic agents (n=24), and benzodiazepines (n=24). Among these, only the use of antihypertensives showed a significant association with fungal infections (*p*=0.009) ( Table 5).

## Discussion

The present study provides a comprehensive analysis of 462 cases of oral viral, fungal, and bacterial infections diagnosed over a 13-year period at a referral center in Brazil. Viral infections, accounting for 65.8% of cases, were the most prevalent, with HPV-induced squamous papilloma as the leading lesion type (75% of viral cases). The high prevalence of HPV-related oral lesions in Brazil highlights a significant public health challenge, reflecting regional variations in genotype distribution and associated risk factors (21). Fungal infections, comprising 29.4% of cases, were predominantly erythematous and pseudomembranous candidiasis and showed associations with advanced age, female sex, and comorbidities such as diabetes and hypertension (6). These features are particularly relevant given Brazil’s aging population and the high prevalence of systemic conditions that impair immunity, increasing susceptibility to fungal infections (22). Bacterial infections, though rare (4.8% of cases), were dominated by actinomycosis, a condition often affecting gnathic bones and mimicking odontogenic infections, which complicates both diagnosis and management (23).

The predominance of HPV-related lesions aligns with global trends; however, the notably high occurrence of squamous papilloma reflects specific aspects of Brazil’s epidemiological profile, where low-risk HPV subtypes are frequently detected in the oral mucosa (21). HPV encompasses a diverse group of over 200 viruses that demonstrate tropism for epithelial tissues in the genital and aerodigestive tracts (24,25). The largest of these, the α subgroup, primarily targets mucosal epithelia, including the oral cavity, where it is responsible for benign epithelial proliferations commonly linked to low-risk genotypes, particularly HPV-6 and HPV-11 (21,25). These genotypes are directly implicated in squamous papilloma development, which emerged as the most prevalent lesion type in our findings. While benign HPV lesions in the oral mucosa often share similar clinical and histopathological features, certain types, such as condyloma acuminatum, are associated with behaviors carrying a higher sexual risk profile (25). This subset of lesions tends to be more common among individuals with multiple sexual partners and may, in certain contexts, act as clinical indicators of sexual abuse (25). This finding emphasizes the need for targeted public health interventions and clinical vigilance in screening for HPV-associated lesions.

Fungal infections, constituting 94.1% of all non-viral infections, were primarily erythematous, chronic hyperplastic, or pseudomembranous candidiasis, consistent with literature emphasizing candidiasis as the most common oral fungal infection (6). These infections often manifested as plaques, pseudomembranes, or macules and were frequently accompanied by burning sensations. Older adults with systemic conditions such as diabetes mellitus and hypertension were particularly vulnerable, with 84.6% of patients with fungal infections reporting xerogenic medication use, such as antihypertensives, and alcohol consumption. Xerogenic drugs reduce salivary flow, creating an environment conducive to fungal overgrowth, a well-documented risk factor for candidiasis (26). Local factors, such as hyposalivation and denture use, further exacerbate this risk, with denture stomatitis affecting up to 70% of denture wearers, depending on hygiene practices (27). Indeed, oral candidiasis in individuals with poorly controlled diabetes are particularly concerning due to the higher prevalence of azole-resistant strains and non-albicans *Candida* species, which often exhibit multidrug resistance (6,9,28,29). While these findings underscore the interplay between systemic health, medication use, and fungal infections, the lack of quantitative data on salivary flow rates and denture maintenance in this study limits further analysis. Future research should incorporate these variables to deepen understanding and improve management strategies for at-risk populations.

Bacterial infections were uncommon in the present study, with actinomycosis accounting for most cases. Actinomycosis, a rare but invasive condition, poses significant diagnostic challenges due to its ability to odontogenic infections (23). Radiographic features such as poorly defined bone destruction often resemble periapical lesions, leading to frequent misdiagnoses. For instance, it has been documented that about 10% of cases in the head and neck region are correctly identified upon initial presentation (23). Immunosuppressed individuals are particularly vulnerable, with untreated cases potentially progressing to life-threatening cervicofacial actinomycosis (23). In the present study, cases of actinomycosis involved the gnathic bones, with lesions closely mimicking periapical conditions, thereby complicating timely diagnosis (23). As previously mentioned, our study did not include other bacterial infections (e.g., periodontitis, pulpal/apical lesions, and Ludwig’s angina), as our tertiary care center primarily addresses non-emergency cases. Such infections are generally managed in primary or emergency care settings in our healthcare system.

This study’s cross-sectional and retrospective design carries inherent limitations. First, the single-center sample may restrict the generalizability of findings, especially in a country as geographically and demographically diverse as Brazil. Regional differences in healthcare access, socioeconomic factors, and institutional practices are likely to influence the epidemiology of oral infections. Second, the absence of data on socioeconomic status, lifestyle factors, and additional confounders limits the ability to analyze broader risk factors comprehensively. Third, reliance on medical records introduces potential biases, including recall and documentation biases, which may affect the accuracy of variables such as symptoms, comorbidities, and treatment histories (30). Despite these shortcomings, the study leverages a large, diverse sample over an extended period, providing valuable insights into the clinicopathological characteristics of oral infections. Future studies should adopt multicenter, prospective designs that integrate socioeconomic and behavioral factors to enhance the epidemiological understanding of these conditions.

In summary, this study identified viral infections as the most prevalent non-odontogenic infections, with squamous papilloma being particularly frequent among men. Fungal infections, notably erythematous and pseudomembranous candidiasis, were prevalent among older women with hypertension, diabetes, xerogenic medication use, and alcohol consumption. Bacterial infections were rare, with actinomycosis as the most frequent, often mimicking odontogenic infections. These findings highlight the importance of considering specific risk factors, including age, comorbidities, and medication use, to improve prevention and management strategies for oral infections.

## Figures and Tables

**Table 1:** Characteristics of the individuals with oral infectious diseases (n=462).

Variable	n (%)
Age (median and range)	49.5 (2–100) years
Sex	
Male	202 (43.7)
Female	260 (56.3)
Evolution time (mean and range)	12.8 (0–2750) months
Viral infection	304 (65.8)
Squamous papilloma	228 (75)
Verruca vulgaris	23 (7.6)
Herpes simplex virus (secondary herpes labialis)	17 (5.6)
Condyloma acuminatum	10 (3.3)
Kaposi's sarcoma (secondary HHV-8)	8 (2.7)
Focal epithelial hyperplasia	8 (2.7)
Plasmablastic lymphoma (secondary HIV)	5 (1.6)
Primary herpetic gingivostomatitis (HHV-1)	1 (0.3)
Herpes zoster	1 (0.3)
Hairy leukoplakia	1 (0.3)
Infectious mononucleosis (secondary EBV)	1 (0.3)
Cytomegalovirus	1 (0.3)
Fungal infection	136 (29.4)
Erythematous candidiasis	96 (70.6)
Pseudomembranous candidiasis	26 (19.1)
Chronic hyperplastic candidiasis	6 (4.4)
Histoplasmosis	3 (2.2)
Mucormycosis	2 (1.5)
Aspergillosis	2 (1.5)
Paracoccidioidomycosis	1 (0.7)
Bacterial infection	22 (4.8)
Actinomycosis	10 (45.4)
Primary syphilis	5 (22.7)
Tuberculosis	5 (22.7)
Secondary syphilis	1 (4.6)
Sinusitis	1 (4.6)

Note: EBV, Epstein-Barr virus; HHV-1, human herpesvirus 1; HHV-8, human herpesvirus-8; HIV, human immunodeficiency virus.

**Table 2:** Association between clinical aspects and signs/symptoms with infectious diseases (n=462).

Variable	Infectious diseases			p value
	Bacterial (n=22)	Fungal (n=136)	Viral (n=304)	
	n (%)	n (%)	n (%)	
Clinical aspects				
Plaque	2 (3.0)	51 (76.1)	14 (20.9)	<0.001*
Ulcer	6 (16.7)	15 (41.7)	15 (41.7)	<0.001**
Nodule	2 (1.3)	6 (3.9)	144 (94.7)	<0.001*
Tumor	2 (25.0)	0 (0.0)	6 (75.0)	0.015*
Vesicle	0 (0.0)	0 (0.0)	2 (100.0)	0.423*
Bullae	0 (0.0)	0 (0.0)	4 (100.0)	0.186*
Fistula	1 (100.0)	0 (0.0)	0 (0.0)	0.057*
Macule	0 (0.0)	45 (84.9)	8 (15.1)	<0.001*
Erosion	0 (0.0)	4 (100.0)	0 (0.0)	0.007*
Petechiae	0 (0.0)	2 (100.0)	0 (0.0)	0.090*
Papule	0 (0.0)	2 (1.9)	103 (98.1)	<0.001*
Signs and symptoms				
Asymptomatic	2 (1.2)	35 (21.3)	127 (77.4)	<0.001*
Pain	6 (11.5)	26 (50.0)	20 (38.5)	<0.001**
Bleeding	1 (16.7)	1 (16.7)	4 (66.7)	0.472*
Drainage	1 (100.0)	0 (0.0)	0 (0.0)	0.057*
Burning	0 (0.0)	54 (93.1)	4 (6.9)	<0.001*
Scaling	0 (0.0)	1 (100.0)	0 (0.0)	0.294*
Xerostomia	0 (0.0)	5 (100.0)	0 (0.0)	0.002*
Pruritus	0 (0.0)	2 (50.0)	2 (50.0)	0.598*
Numbness	0 (0.0)	0 (0.0)	1 (100.0)	0.658*
Suppuration	0 (0.0)	1 (100.0)	0 (0.0)	0.294*
Edema	0 (0.0)	0 (0.0)	2 (100.0)	0.432*

Note: *Likelihood ratio test; **Pearson’s Chi-square test.

**Table 3:** Association between sociodemographic variables, evolution time, harmful habits, anatomical location with infectious diseases (n=462).

Variables	Infectious diseases			p value
	Bacterial (n=22)	Fungal (n=136)	Viral (n=304)	
	n (%)	n (%)	n (%)	
Sex				
Male	10 (5.0)	36 (17.8)	156 (77.2)	<0.001^**^
Female	12 (4.6)	100 (38.5)	148 (56.9)	
Age (mean and SD)	41.8 ± 14.4 years^a^	59 ± 16.4 years^b^	42.3 ± 20.0 years^a^	<0.001^*^
Evolution time (mean and SD)	5.4 ± 15.5 months	6.8 ± 17.1 months	16 ± 158.3 months	0.267^*^
Smoking				
No	21 (5.1)	121 (29.3)	271 (65.6)	0.575^***^
Yes	1 (2.0)	15 (30.6)	33 (67.3)	
Alcoholism				
No	22 (5.1)	122 (28.2)	288 (66.7)	0.038^***^
Yes	0 (0.0)	14 (46.7)	16 (53.3)	
Anatomical location^#^				
Oropharynx	0 (0.0)	0 (0.0)	2 (100.0)	0.432^***^
Tongue	6 (3.9)	54 (34.8)	95 (61.3)	0.181^**^
Buccal mucosa	2 (3.9)	19 (37.3)	30 (58.8)	0.441^***^
Lips	1 (5.3)	5 (7.2)	63 (91.3)	<0.001^***^
Soft palate	1 (2.7)	1 (2.7)	35 (94.6)	<0.001^***^
Hard palate	2 (2.0)	62 (59.0)	41 (39.0)	<0.001^***^
Alveolar ridge	1 (5.6)	11 (61.1)	6 (33.3)	0.014^***^
Labial commissure	0 (0.0)	7 (33.3)	14 (66.7)	0.340^***^
Floor of the mouth	0 (0.0)	1 (8.3)	11 (91.7)	0.088^***^
Gingiva	0 (0.0)	2 (6.9)	27 (93.1)	0.001^***^
Vestibular sulcus	0 (0.0)	1 (50.0)	1 (50.0)	0.774^***^
Retromolar region	0 (0.0)	1 (100.0)	0 (0.0)	0.294^***^
Maxilla (mucosa, NS)	7 (70.0)	1 (10.0)	2 (20.0)	<0.001^***^
Mandible (mucosa, NS)	4 (80.0)	1 (20.0)	0 (0.0)	<0.001^***^

Note: NS, not specified; SD, standard deviation. #The unit of analysis of the variable anatomical location was not the number of individuals, but the number of lesions presented. *Kruskall-Wallis test with Dunn’s post-hoc Mann-Whitney test (different letters in the same row indicate *p*-values <0.05). **Pearson’s Chi-square test. ***Likelihood ratio test.

**Table 4:** Association of comorbidities in individuals with oral infectious diseases (n=177)*.

Variable	Infectious diseases (n, %)			p value^*^
	Bacterial (n=2)	Fungal (n=206)	Viral (n=38)	
Hepatitis C	0 (0.0)	1 (50.0)	1 (50.0)	0.649
Liver disease	0 (0.0)	2 (100.0)	0 (0.0)	0.513
Pneumonia (not specified)	0 (0.0)	1 (100.0)	0 (0.0)	0.717
Hyperthyroidism	1 (50.0)	1 (50.0)	0 (0.0)	0.139
Hypothyroidism	0 (0.0)	12 (75.0)	4 (25.0)	0.416
Hypertension	1 (1.5)	56 (84.8)	9 (13.6)	0.006
Depression	0 (0.0)	14 (93.3)	1 (6.7)	0.075
Anxiety	0 (0.0)	13 (86.7)	2 (13.3)	0.238
Scleroderma	0 (0.0)	3 (100.0)	0 (0.0)	0.366
Genital herpes	0 (0.0)	2 (100.0)	0 (0.0)	0.513
Rheumatoid arthritis	0 (0.0)	3 (75.0)	1 (25.0)	0.809
Fibromyalgia	0 (0.0)	3 (100.0)	0 (0.0)	0.366
Diabetes mellitus	0 (0.0)	25 (89.3)	3 (10.7)	0.028
Heart disease	0 (0.0)	8 (72.7)	3 (27.3)	0.539
People living with HIV	0 (0.0)	3 (33.3)	6 (66.7)	0.013
Nephropathy (not specified)	0 (0.0)	7 (87.5)	1 (12.5)	0.450
Chronic kidney failure	0 (0.0)	1 (50.0)	1 (50.0)	0.649
Thrombocytopenic purpura	0 (0.0)	1 (100.0)	0 (0.0)	0.717
Patellar chondropathy	0 (0.0)	1 (100.0)	0 (0.0)	0.717
Atopic dermatitis	0 (0.0)	1 (100.0)	0 (0.0)	0.717
Dystonia	0 (0.0)	1 (100.0)	0 (0.0)	0.717
Epilepsy	0 (0.0)	1 (100.0)	0 (0.0)	0.717
Squamous cell carcinoma	0 (0.0)	2 (66.7)	1 (33.3)	0.803
Glaucoma	0 (0.0)	5 (100.0)	0 (0.0)	0.185
Hodgkin's lymphoma	0 (0.0)	0 (0.0)	1 (100.0)	0.229
Melanoma	0 (0.0)	1 (100.0)	0 (0.0)	0.717
Myopathy	0 (0.0)	1 (100.0)	0 (0.0)	0.717
Osteoarthritis	0 (0.0)	7 (87.5)	1 (12.5)	0.450
Osteopenia	0 (0.0)	2 (100.0)	0 (0.0)	0.513
Osteoporosis	0 (0.0)	2 (100.0)	0 (0.0)	0.513
Thrombosis (not specified)	0 (0.0)	3 (100.0)	0 (0.0)	0.366
Anemia	0 (0.0)	2 (66.7)	1 (33.3)	0.803
Chronic obstructive pulmonary disease	0 (0.0)	7 (77.8)	2 (22.2)	0.610
Colorectal cancer	0 (0.0)	2 (100.0)	0 (0.0)	0.513
Colitis (not specified)	0 (0.0)	1 (100.0)	0 (0.0)	0.717
Labyrinthitis	0 (0.0)	4 (100.0)	0 (0.0)	0.260
Neuropathy (not specified)	0 (0.0)	3 (100.0)	0 (0.0)	0.366
Systemic lupus erythematosus	0 (0.0)	1 (100.0)	0 (0.0)	0.717
Sjögren disease	0 (0.0)	1 (100.0)	0 (0.0)	0.717
Stevens-Johnson syndrome	0 (0.0)	2 (100.0)	0 (0.0)	0.513

**Table 5:** Association of medications used with oral infectious diseases (n=177).

Variable	Infectious diseases			p-value^*^
	Bacterial (n=3)	Fungal (n=261)	Viral (n=49)	
	n (%)	n (%)	n (%)	
Antihypertensive	1 (1.5)	55 (84.6)	9 (13.8)	0.009
Hypoglycemic	0 (0.0)	21 (87.5)	3 (12.5)	0.077
Vitamin Supplementation	0 (0.0)	14 (93.3)	1 (6.7)	0.075
Nonsteroidal anti-inflammatory	0 (0.0)	3 (42.9)	4 (57.1)	0.115
Antibiotic	0 (0.0)	1 (25.0)	3 (75.0)	0.079
Glucocorticoid	0 (0.0)	4 (100.0)	0 (0.0)	0.260
Antifungal	0 (0.0)	4 (66.7)	2 (33.3)	0.641
Hormone Replacement	1 (5.9)	14 (82.4)	2 (11.8)	0.456
Antiphysical	0 (0.0)	1 (100.0)	0 (0.0)	0.717
Anticoagulant	0 (0.0)	14 (93.3)	1 (6.7)	0.075
Antiviral	0 (0.0)	1 (100.0)	0 (0.0)	0.717
Gastric Protector	0 (0.0)	24 (88.9)	3 (11.1)	0.085
Statins	0 (0.0)	19 (82.6)	4 (17.4)	0.189
Antiarrhythmics	0 (0.0)	14 (87.5)	2 (12.5)	0.192
Topical corticosteroids	0 (0.0)	1 (100.0)	0 (0.0)	0.717
Benzodiazepines	0 (0.0)	21 (87.5)	3 (12.5)	0.077
Antihistaminic	0 (0.0)	5 (83.3)	1 (16.7)	0.652
Bisphosphonates	0 (0.0)	2 (100.0)	0 (0.0)	0.513
Antiepileptic	0 (0.0)	11 (91.7)	1 (8.3)	0.172
Antimalarial	0 (0.0)	2 (100.0)	0 (0.0)	0.513
Antiemetic	0 (0.0)	3 (75.0)	1 (25.0)	0.809
Bronchodilator	0 (0.0)	2 (100.0)	0 (0.0)	0.513
Antineoplastic	0 (0.0)	1 (100.0)	0 (0.0)	0.717
Immunosuppressants	0 (0.0)	2 (66.7)	1 (33.3)	0.803
Immunomodulator	0 (0.0)	0 (0.0)	1 (100.0)	0.229
Haart	0 (0.0)	3 (50.0)	3 (50.0)	0.281
Cholinesterase Inhibitor	0 (0.0)	1 (100.0)	0 (0.0)	0.717
Antiasthmatic	0 (0.0)	1 (100.0)	0 (0.0)	0.717
Analgesic	0 (0.0)	2 (66.7)	1 (33.3)	0.803
Antispasmodic	0 (0.0)	1 (100.0)	0 (0.0)	0.717
Anticholinergics	0 (0.0)	1 (100.0)	0 (0.0)	0.717
Antidepressant	1 (5.9)	13 (76.5)	3 (17.6)	0.841

Note: *Likelihood Ratio test.

## Data Availability

The datasets used and/or analyzed during the current study are available from the corresponding author.
